# Yellow-necked mice (*Apodemus flavicollis*) and bank voles (*Myodes glareolus*) as zoomonitors of environmental contamination at a polluted area in Slovakia

**DOI:** 10.1186/1751-0147-52-58

**Published:** 2010-11-05

**Authors:** Monika Martiniaková, Radoslav Omelka, Birgit Grosskopf, Alena Jančová

**Affiliations:** 1 Department of Zoology and Anthropology, Constantine the Philosopher University, Nábrežie mládeže 91, 949 74 Nitra, Slovak Republic; 2 Department of Botany and Genetics, Constantine the Philosopher University, Nábrežie mládeže 91, 949 74 Nitra, Slovak Republic; 3 Johann Friedrich Blumenbach Institute of Zoology and Anthropology, Georg-August University, Bürgerstrasse 50, 37 073 Göttingen, Germany

## Abstract

**Background:**

Free-living wild rodents are often used as zoomonitors of environmental contamination. In the present study, accumulation of cadmium (Cd), copper (Cu), iron (Fe), and zinc (Zn) in critical organs of yellow-necked mice (*Apodemus flavicollis*) and bank voles (*Myodes glareolus*) trapped in a polluted area in Nováky, Slovakia was investigated.

**Methods:**

Yellow-necked mice (n = 8) and bank voles (n = 10) were collected using standard theriological methods for wood ecosystems. All animals were adult males in good physical condition. The concentrations of Cd, Cu, Fe, and Zn in the liver, kidney, and bone were determined by atomic absorption spectrophotometry.

**Results:**

The highest concentrations of Cd and Zn were found in the bone of both species while Cu and Fe accumulated mainly in kidney or liver. Significant higher concentrations of Cd and Cu were detected in the liver of bank voles than in yellow-necked mice. Similar significant higher levels of Cd and Zn were found in the bone of bank voles. In contrast, significant higher concentrations of Cu and Fe were present in the kidney of yellow-necked mice.

**Conclusions:**

In the yellow-necked mouse and bank vole, bone seems to accumulate Cd and Zn following prolonged exposure. On the contrary, kidney and liver store Cu and Fe after a long-term environmental exposure. In the present study, bank voles seemed to be more heavy metal loaded zoomonitors than yellow-necked mice.

## Background

The importance of monitoring the exposure and studying the effects of heavy metals on living organisms has increased in the last decades. Studies of small mammals, mainly free-living wild rodents, have demonstrated an ability to accumulate a wide spectrum of pollutants [1,2]. Significant relations have been found between residues of metals in soil and in organs or tissues [1,3]. In addition, the patterns of heavy metal distribution in rodent tissues and their concentrations are similar to those found in humans. Therefore, rodents frequently serve as models for humans in ecotoxicology [4]. Free-living wild rodents are suitable for monitoring environmental pollution and exposure risk for people living in a contaminated area [5,6].

Mice of the genus *Apodemus *and voles are suitable pollution zoomonitors [7-9]. The yellow-necked mouse (*Apodemus flavicollis*) and bank vole *(Myodes glareolus*; formerly *Clethrionomys glareolus*) belong to the most dominant rodent species in Slovakia. These animals are easily caught and they have a small migration area and a relatively short life span. Compared to larger mammals, their higher metabolic rate may increase their susceptibility to pollutants. Among heavy metals causing environmental contamination, cadmium (Cd) is among the most dangerous metals. This non-essential metal is toxic for humans or animals even in very low concentrations [10]. It primarily damages kidney, lung, and bones, e.g. through altered calcium metabolism leading to osteomalacia [11]. Copper (Cu), iron (Fe), and zinc (Zn) are among the physiologically important metals that although being essential, may induce toxic effects if provided in high concentrations [12]. The aim of the present study was to determine concentrations of Cd, Cu, Fe and Zn in the liver, kidney and bone of yellow-necked mice and bank voles trapped in a polluted area in Nováky, Slovakia.

## Methods

### Animals

**Figure 1:**
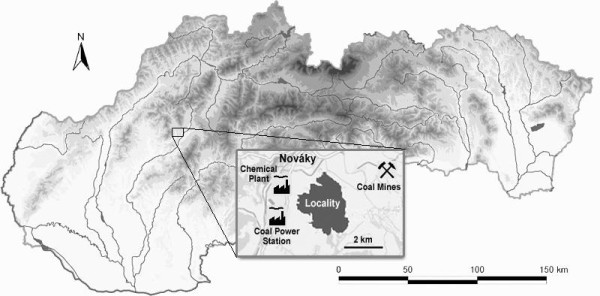
**Map of investigated polluted area in Slovakia**.

Yellow-necked mice (n = 8) and bank voles (n = 10) were obtained by means of the standard theriological methods and procedures for wood ecosystems [13] in February 2007. The rodents were trapped in a polluted area in Nováky, Prievidza district, Slovakia, which is considered as a heavily polluted region. Possible sources of pollution in this region are the Nováky chemical plant, the coal power station in Nováky, and Handlová - Cígeľ coal mines (Figure 1). All animals caught were adult males (aged 4-5 months determined by dental wear). They appeared in good physical condition and without gross lesions at necropsy.

### Procedures

The animals were euthanized by cervical dislocation shortly after capture and examined for gross lesions. Samples of liver, kidney, and bone (femur) were kept at -18 °C until analysis. The concentrations of Cd, Cu, Fe, and Zn were determined by atomic absorption spectrophotometry (Perkin Elmer 4100 ZL) in a graphite furnace [14]. Samples of liver and kidney were weighed and ashed with diluted nitric acid p.a. (HNO_3_:H_2_O = 2:1) at 130°C for 2 h. Undissolved particles were filtered off and the solution diluted to 25 ml [15]. The bone samples were dried at 105°C until dry mass was obtained. Then all samples were weighed (minimum 2 g) and digested in concentrated nitric acid at 90°C for 10 h. The samples were diluted to 25 ml with distilled water before analysis [16]. Detection limits were as follows Cd = 0.005 ppm, Cu = 0.01 ppm, Fe = 0.02 ppm, and Zn = 0.13 ppm. The recovery of the method was 96-98% and reproducibility was better than 1.0%. All metal concentrations were expressed on a dry weight basis in mg.kg^-1^.

### Statistics

From the final data, basic statistical characteristics were calculated (mean, standard deviation, minimum, maximum, median). Since the distribution of observed levels of heavy metals was normal according to the Shapiro-Wilk test, the parametric Student's t test was used for species comparisons employing the Statistica 7.0 software program.

## Results

Concentrations of Cd, Cu, Fe, and Zn in the liver, kidney, and bone of the examined yellow-necked mice and bank voles are listed in Table 1. In both species, the highest concentrations of Cd were found in bone followed by kidney and liver. The hierarchy of Cu and Fe concentrations was kidney > liver > bone in yellow-necked mice. In the bank vole, highest concentration of Cu was detected in the liver followed by bone and kidney while the hierarchy for Fe concentrations was liver > kidney > bone. In both species, the highest concentrations of Zn were found in the bone followed by liver and kidney.

**Table 1:** Heavy metal concentrations

Tissue	Species		Cd (mg.kg^-1^)	Cu (mg.kg^-1^)	Fe (mg.kg^-1^)	Zn (mg.kg^-1^)
Liver	Yellow-necked mouse	x	0.023	5.13	455.23	101.06
		
		sd	0.004	1.79	192.39	40.19
		
		min	0.019	3.24	276.10	55.47
		
		max	0.027	6.69	658.60	167.60
		
		med	0.023	5.77	431.00	95.31
	
	Bank vole	x	0.047*	7.26*	461.77	175.35
		
		sd	0.027	2.96	77.86	159.25
		
		min	0.031	3.96	388.20	66.15
		
		max	0.078	9.65	543.30	358.20
		
		med	0.032	8.17	453.80	101.70

Kidney	Yellow-necked mouse	x	0.071	5.38*	506.90*	81.64
		
		sd	0.031	1.20	65.57	53.38
		
		min	0.035	3.99	434.50	47.43
		
		max	0.094	6.22	562.30	200.00
		
		med	0.083	5.92	523.90	62.19
	
	Bank vole	x	0.075	3.05	394.73	80.28
		
		sd	0.039	0.98	95.54	36.35
		
		min	0.031	1.91	287.70	55.38
		
		max	0.104	3.66	471.40	122.00
		
		med	0.089	3.57	425.10	63.46

Bone	Yellow-necked mouse	x	2.53	3.60	156.61	126.88
		
		sd	0.77	0.47	31.64	10.35
		
		min	1.93	2.89	115.98	110.96
		
		max	3.95	4.27	204.45	141.35
		
		med	2.76	3.81	168.57	129.14
	
	Bank vole	x	4.61*	3.78	138.98	176.49*
		
		sd	1.13	0.74	10.15	11.20
		
		min	3.71	3.20	128.19	164.21
		
		max	5.88	4.61	140.42	186.14
		
		med	3.76	3.81	138.17	174.14

x - mean, sd - standard deviation, min - minimum, max - maximum, med - median, (*) - *P *< 0.05

Concentrations of cadmium (Cd), copper (Cu), iron (Fe) and zinc (Zn) in the liver, kidney and bone (femur) of yellow-necked mouse (*Apodemus flavicollis*) (n = 8) and bank vole (*Myodes glareolus*) (n = 10).

When comparing heavy metal levels in the two rodent species, significant higher concentrations of Cd and Cu were detected in the liver of the bank vole than in the yellow-necked mouse (*P *< 0.05). Significant higher levels of Cd and Zn were present in the bone tissue of the bank vole (*P *< 0.05) than in the yellow-necked mouse, while these mice had significant higher kidney concentrations of Cu and Fe (P < 0.05).

## Discussion

Previous studies have demonstrated that the coal power station in Nováky and the Nováky chemical plant have negative effects on the environment especially by soil pollution [17] and water pollution [18]. One of the most important sources of environmental contamination with heavy metals is the coal industry [19,20]. The dust emitted from this kind of industries contains Cd, lead (Pb), Cu and Zn, and the associated environmental contamination may increase the heavy metal content of mammals inhabiting the polluted areas.

In general, there is a significant relationship between the amount of heavy metals in the environment and in the organs of free-living wild rodents, first of all in liver and kidneys [13]. However, some metals e.g. Pb, accumulate mainly in bone. Bone tissue has some advantages compared with soft tissues in ecotoxicological studies as metals are subjected to the rather slow bone turnover (approximately 10%/y in adult individuals). Therefore, an accurate historic record of exposure to various elements is retained in the bone and consequently, bone tissue is a suitable bioindicator of a long-term environmental exposure [16].

We found higher concentrations of Zn in the liver, kidney and bone of bank voles than Milton *et al. *[21], who determined Pb, Zn, and Cd concentrations in selected organs of bank voles trapped at the contaminated abandoned Pb mine at Frongoch in west Wales. The hierarchy of Zn concentrations in their study was bone > liver > kidney > muscle. The same hierarchy was also observed in our study. According to Milton *et al. *[21], the hierarchy of Cd concentrations in the tissues was kidney > bone > liver > muscle. In our study, the highest concentration of Cd was detected in the bone of bank vole followed by liver and kidney. In addition, Cd concentration in the bone was higher than found by Milton *et al. *[21]. These data demonstrate increased accumulation of Zn, Cd in critical organs of bank voles from Nováky and thus provide further evidence of intensive environmental pollution of this area. Since distribution and levels of heavy metals in soft and hard tissues of free-living rodents are similar to those found in humans [5,6], it is believed that the same accumulation of Cd, and Zn occurs also in humans living in studied area of Slovakia. In yellow-necked mice and bank voles, bone accumulates highest levels of Cd and Zn after long-term environmental exposure. On the contrary, Cu and Fe accumulated mainly in kidney or liver of both rodent species.

According to Pokarzhevskij [22], the concentration in the body of a given element is practically directly proportional to its amount in the food. Since the age of the rodents studied was 4-5 months, they foraged on the autumn and winter spectrum of food, including beechnuts and acorns in yellow-necked mouse, and berries, fungi, large amounts of grass leaves in bank vole [23]. Sawicka-Kapusta *et al. *[24] have recorded that Cd, Pb, Cu and Zn concentrations in yellow-necked mice are significantly lower than those in bank voles. The same correlations have been established in the study by Metcheva *et al. *[7] who detected heavy metal concentration in the liver and body of rodent species from different Bulgarian regions. In our study, significant higher concentrations of Cd and Cu were detected in the liver of bank voles than in yellow-necked mice. Also, higher levels of Cd and Zn were found in the bone of this species. In the kidney of yellow-necked mice, significant higher concentrations of Cu and Fe were present, possibly due to lower renal excretion rates for these metals in yellow-necked mice.

In general, it is known that differences in average metal concentrations between species can be the result of differences in population structure between the species. In addition, the metal concentrations in free-living rodents may be affected by altered feeding patterns, seasonal and flood-related aspects of food availability, habitat suitability and connectivity, and life-stage-related food preference combined with variations in the metal contents in the food items themselves. Finally, exposure time, and therefore age of the animals, might be an explanatory factor [2]. Taking into account all these aspects, we suppose that the bank vole is a more heavy metal loaded zoomonitor than the yellow-necked mouse.

## Conclusions

Highest concentrations of Cd and Zn were found in the bone of both yellow-necked mice and bank voles. Cu and Fe accumulated mainly in kidney or liver. Significant higher concentrations of Cd and Cu were detected in the liver of bank vole. In the bone of this species, significant higher levels of Cd and Zn were also found. Significant higher concentrations of Cu and Fe were present in the kidney of yellow-necked mouse. Bank vole is considered as a more pollution loaded zoomonitor in comparison with yellow-necked mouse.

## Competing interests

The authors declare that they have no competing interests.

## Authors' contributions

MM was responsible for animal trapping and determination of heavy metals concentrations of bones. RO was responsible for the statistical analyses. BG was responsible for sample preparation for atomic absorption spectrophotometry. AJ was responsible for analyses of liver and kidneys. All authors read and approved the final manuscript.

## References

[B1] Ieradi LA Moreno S Bolívar JP Cappai A Di Benedetto A Cristaldi M Free-living rodents as bioindicators of genetic risk in natural protected areas Environ Pollut 1998 102 265 268 10.1016/S0269-7491(98)00077-3

[B2] Wijnhoven S Leuven RSEW Van der Velde G Jungheim G Koelemij EI de Vries FT Eijsackers HJP Smits AJM Heavy-metal concentrations in small mammals from a diffusely polluted floodplain: importance of species- and location-specific characteristics Arch Environ Contam Toxicol 2007 52 603 613 10.1007/s00244-006-0124-1 17387425 PMC1914299

[B3] Shore RF Predicting cadmium, lead and fluoride levels in small mammals from soil residues and by species-species extrapolations Environ Pollut 1995 88 333 340 10.1016/0269-7491(95)93447-8 15091546

[B4] Shore RF Rattner BA Ecotoxicology of wild mammals 2001 London: John Wiley & Sons

[B5] O'Brien DJ Kaneene JB Poppenga RH The use of mammals as sentinels for human exposure to toxic contaminants in the environment Environ Health Perspect 1993 99 351 368 10.2307/3431509 8319652 PMC1567056

[B6] Damek-Poprawa M Sawicka-Kapusta K Damage to the liver, kidney, and testis with reference to burden of heavy metals in yellow-necked mice from areas around steelworks and zinc smelters in Poland Toxicology 2003 186 1 10 10.1016/S0300-483X(02)00595-4 12604166

[B7] Metcheva R Teodorova S Topashka-Ancheva M A comparative analysis of the heavy metals and toxic elements loading indicated by small mammals in different Bulgarian regions Acta Zool Bulg 2001 53 61 80

[B8] Milton A Johnson MS Cooke JA Lead within ecosystems on metalliferous mine tailings in Wales and Ireland Sci Total Environ 2002 299 177 190 10.1016/S0048-9697(02)00253-X 12462584

[B9] Milton A Cooke JA Johnson MS A comparison of cadmium in ecosystems on metalliferous mine tailings in Wales and Ireland Water Air Soil Pollut 2004 153 157 172 10.1023/B:WATE.0000019940.76065.21

[B10] Haider S Naithani V Barthwal J Kakkar P Heavy metal content in some therapeutically important medicinal plants Bull Environ Contam Toxicol 2004 72 119 127 10.1007/s00128-003-0249-0 15058663

[B11] Kido T Nogawa K Hochi Y Hayano M Honda R Tsuritani I Ishizaki M The renal handling of calcium and phosphorus in environmental cadmium-exposed subjects with renal dysfunction J Appl Toxicol 1993 13 43 47 10.1002/jat.2550130110 8440874

[B12] Angelova V Ivanova R Delibaltova V Ivanov K Bio-accumulation and distribution of heavy metals in fiber crops (flax, cotton, and hemp) Ind Crops Prod 2004 19 197 205 10.1016/j.indcrop.2003.10.001

[B13] Jančová A Massányi P Naď P Koréneková B Skalická M Drábeková J Baláž I Accumulation of heavy metals in selected organs of yellow necked mouse *(Apodemus flavicollis)* Ekol Bratislava 2006 25 19 26

[B14] Stawarz R Zakrzewski M Marenčík A Hraška Š Heavy metal concentration in the toad *Bufo Bufo *from a region of Mochovce, Slovakia Ekol Bratislava 2003 22 292 297

[B15] Kramárová M Massányi P Jančová A Toman R Slamečka J Tataruch F Kováčik J Gašparík P Naď P Skalická M Koréneková B Jurčík R Čuboň J Haščík P Concentration of cadmium in liver and kidneys of some wild and farm animals Bull Vet Inst Pulawy 2005 49 465 469

[B16] Martiniaková M Omelka R Jančová A Stawarz R Formicki G Concentrations of selected heavy metals in bones and femoral bone structure of bank (*Myodes glareolus*) and common (*Microtus arvalis*) voles from different polluted biotopes in Slovakia Arch Environ Contam Toxicol 2010 in press 10.1007/s00244-010-9545-y PMC3047683 20532880

[B17] Keegan T Hong B Thornton I Farago M Jakubis P Pesch B Ranft U Nieuwenhuijsen MJ Expascan Study Group Assessment of environmental arsenic levels in Prievidza district J Expo Anal Environ Epidemiol 2002 12 179 185 10.1038/sj.jea.7500216 12032814

[B18] Labunska I Brigden K Santillo D Stringer R The Nováky chemical plant (Novácke chemické závody) as a source of mercury and organochlorine contaminants to the Nitra river, Slovakia 2002 Exeter: Greenpeace Research Laboratories

[B19] Ieradi LA Zima J Allegra F Kotlánová E Campanella L Grossi R Cristaldi M Evaluation of genotoxic damage in wild rodents from a polluted area in the Czech Republic Folia Zool 2003 52 57 66

[B20] Roberts RD Johnson MS Dispersal of heavy metals from abandoned mine transference through terrestrial food chains Environ Pollut 1978 16 293 310 10.1016/0013-9327(78)90080-0

[B21] Milton A Cooke JA Johnson MS Accumulation of lead, zinc, and cadmium in a wild population of *Clethrionomys glareolus *from an abandoned lead mine Arch Environ Contam Toxicol 2003 44 405 411 10.1007/s00244-002-2014-5 12712302

[B22] Pokarzhevskij AD Geochemical ecology of terrestrial animals 1985 Moscow: Nauka Publ House

[B23] Abt KF Bock WF Seasonal variations of diet composition in farmland field mice *Apodemus spp*. and bank voles *Clethrionomys glareolus* Acta Theriol 1998 43 379 389

[B24] Sawicka-Kapusta K Górecki A Lange R Heavy metals in rodents from polluted forests in southern Poland Ekologia Polska 1987 35 345 354

